# A New Approach to Standardize Multicenter Studies: Mobile Lab Technology for the German Environmental Specimen Bank

**DOI:** 10.1371/journal.pone.0105401

**Published:** 2014-08-20

**Authors:** Dominik Lermen, Daniel Schmitt, Martina Bartel-Steinbach, Christa Schröter-Kermani, Marike Kolossa-Gehring, Hagen von Briesen, Heiko Zimmermann

**Affiliations:** 1 Department of Cell Biology & Applied Virology, Fraunhofer-Institute for Biomedical Engineering, St. Ingbert, Saarland, Germany; 2 Department of Laboratory & Information Technology, Fraunhofer-Institute for Biomedical Engineering, St. Ingbert, Saarland, Germany; 3 Federal Environment Agency (UBA), Berlin, Berlin, Germany; 4 Saarland University, Saarbruecken, Saarland, Germany; University of Cape Town, South Africa

## Abstract

Technical progress has simplified tasks in lab diagnosis and improved quality of test results. Errors occurring during the pre-analytical phase have more negative impact on the quality of test results than errors encountered during the total analytical process. Different infrastructures of sampling sites can highly influence the quality of samples and therewith of analytical results. Annually the German Environmental Specimen Bank (ESB) collects, characterizes, and stores blood, plasma, and urine samples of 120–150 volunteers each on four different sampling sites in Germany. Overarching goal is to investigate the exposure to environmental pollutants of non-occupational exposed young adults combining human biomonitoring with questionnaire data. We investigated the requirements of the study and the possibility to realize a highly standardized sampling procedure on a mobile platform in order to increase the required quality of the pre-analytical phase. The results lead to the development of a mobile epidemiologic laboratory (epiLab) in the project “Labor der Zukunft” (future’s lab technology). This laboratory includes a 14.7 m^2^ reception area to record medical history and exposure-relevant behavior, a 21.1 m^2^ examination room to record dental fillings and for blood withdrawal, a 15.5 m^2^ biological safety level 2 laboratory to process and analyze samples on site including a 2.8 m^2^ personnel lock and a 3.6 m^2^ cryofacility to immediately freeze samples. Frozen samples can be transferred to their final destination within the vehicle without breaking the cold chain. To our knowledge, we herewith describe for the first time the implementation of a biological safety laboratory (BSL) 2 lab and an epidemiologic unit on a single mobile platform. Since 2013 we have been collecting up to 15.000 individual human samples annually under highly standardized conditions using the mobile laboratory. Characterized and free of alterations they are kept ready for retrospective analyses in their final archive, the German ESB.

## Introduction

Multicenter sampling events are the cornerstone of a high diversity of epidemiologic studies, health related environmental monitoring, and human biomonitoring studies [1]–[8]. These studies have an increasing demand for standardized working conditions and a standardized pre-analytical phase since misleading results due to improper sampling conditions are currently more relevant than errors occurring during lab analysis [9]–[14]. Once a sample is collected in an improper way the best analytical tool will not be able to reveal its pristine information. Errors in the pre-analytical phase mostly occur at the time of specimen collection. According to Bonini and colleagues (2002) errors in the pre-analytical phase predominate in the laboratory, ranging from 31.6% to 75% compared to errors that may occur in the analytical or post-analytical phase [15]–[19].

To reduce errors in the pre-analytical phase of multicenter sampling events, mobile units can provide the required infrastructure on various sampling sites and therewith simplify standardization. Mobile units based on diverse vehicle platforms are known from both the medical field and environmental research. Medical applications are mainly dedicated to acute medicine, thus ambulances equipped with specific instruments for the investigation of diseases like myocardial infarction [20], [21], lung cancer [22], and stroke [23], [24]. Mobile units are favorably used in natural disaster scenarios [25], [26], during the outbreak of severe infectious diseases [27], and in military campaigns [28]. However, the latter examples show that more advanced mobile medical units for interventions are rather based on containers than on vans or trailers. Medical laboratories “on wheels” are only reported for restricted analytical procedures [1] and in the field of human health monitoring programs [29]. Environmental research is utilizing mobile units for on-site analysis of water and soil [30]. Specific units have been used to determine atmospheric pollutants [31]–[34] and xenobiotics [35]. Interestingly, samples are mostly analyzed on-site and sample storage is of minor concern.

The German ESB, as one example of a multicenter study in Germany, is a central element of the German environmental monitoring system. It is an archive of samples from representative animals and plants, soil, suspended particles, and human samples like blood, plasma, 24 h-urine, hair, and saliva (http://www.umweltprobenbank.de/en/). The German ESB collects, cryopreserves, cryostores, and analyzes human samples since the early 1980s [36], [37]. Every year a maximum of 15.000 human samples from up to 600 young adults at four sites in Germany (Muenster, Halle/Saale, Greifswald, Ulm) are being collected. The blood, plasma and 24 h-urine samples subsequently get analyzed on selected environmental pollutants and physiological parameters. Stored samples allow rapid retrospective monitoring of emerging contaminants whenever needed. Since it is not known which chemicals will be of interest in the future and which concentrations of these chemicals can be found in environmental or human samples, it is highly important to avoid contaminations during the sampling procedure and during the pre-analytical phase. During sampling dental status is recorded. Medical history, exposure-relevant behavior, dietary habits, and living conditions of each volunteer are documented, using a standardized and self-reported questionnaire. Analytical results and data resulting from the questionnaire of each volunteer are statistically evaluated and interpreted, and afterwards reported to the Federal Environment Agency (UBA) and the Federal Ministry for the Environment, Nature Conservation, Building and Nuclear Safety (BMUB) on an annual basis. Thereby, the German ESB generates important information on internal exposures of humans and provides a scientific basis to decide on the necessity of risk reduction measures to protect human health and the environment as well as to control their success. Recent examples from the German ESB human related work are retrospective analyses of heavy metals, Hexamoll, DINCH, Bisphenol A, phthalates and perfluorinated compounds in body fluids [6], [38]–[42].

The sampling of human samples for the German ESB was conducted in the facilities of collaborative institutes and universities in 2012 and the years before. For the routine implementation of the sampling process, a specific infrastructure considering the requirements of acts and regulations on biological safety and quality assurance was required and set up at each site. At least five separated rooms, including a reception, two diagnostic areas, a BSL 2 laboratory, a waiting room, and a van for transportation of samples with a complete cryo-equipment and an oxygen monitoring system were needed. For all processes of the sampling, SOPs were established by the German ESB (http://www.umweltprobenbank.de/de/documents/10022). For each sampling site these SOPs had to be adapted to the specific conditions of the site. Besides, all members of staff had to be trained specifically according to those SOPs. Having this in mind, the question arose in how far it would be possible to integrate a BSL 2 laboratory and the required infrastructure on a single mobile platform. Hence, the goal of this study was to identify the requirements for the implementation of a mobile biosafety level 2 laboratory and to evaluate its feasibility in a routine sampling in 2013.

## Materials and Methods

### Ethical statement

The study protocol of the German ESB was approved by the ethics committee of the Medical Association Saarland, Germany. The positive vote was made available to the partners of the sampling areas Muenster, Halle/Saale, Greifswald, and Ulm for submission to the local ethics committees. All study participants gave written informed consent on standardized forms approved by the same ethics committee. The right to know or not to know was guaranteed and records on the investigation results were supplied to the participants immediately after the analyses of the samples were completed.

### Process and infrastructure of the routine sampling in 2012

#### Acquisition of participants and the pre-sampling phase

At each sampling site a maximum of 75 female and 75 male healthy students between 20 and 29 years were recruited. Volunteers were asked to register via an online registration form and to choose a defined date for blood withdrawal (https://umweltprobenbank.fraunhofer.de). Subsequently, every volunteer received a parcel with a container for collecting the 24 h-urine and an information kit. This kit contained general information about the processes and goals of the German ESB, specific information about the types of samples, the sampling procedure, and an instruction on how to collect the 24 h-urine. Furthermore, a material transfer agreement, two copies of a consent form, information on medical confidentiality and protection of privacy, and a 10-page standardized self-reported questionnaire to record medical history, individual behavior, and potential exposures were included. Volunteers were asked to fill in the questionnaire, to collect their 24 h-urine, to sign the consent form and to bring all of these items to their chosen appointment.

#### Medical history, exposure-relevant behavior, socio-demographic data and incentives

At the sampling site the volunteers handed over the described documents. Every questionnaire was checked for completeness and plausibility to increase data quality. The urine samples were directly transferred into the laboratory for further processing. The questionnaire was linked to the volunteers sample via a respective anonymous number. An allowance was paid to every volunteer after the sampling was finished. Two staff members and two separated desks were required for the implementation of the reception of 75 volunteers per day. A room of at least 8 m^2^ with two separate cabins was needed for this first stage of the sampling process.

#### Dental fillings

For the evaluation of the internal exposure to chemicals (such as mercury or bisphenol A) released from dental fillings, their number and size were recorded for every volunteer by a dentist and one assistant. Dental fillings were recorded following the respective SOP. A separate room of at least 6 m^2^ was equipped with a treatment chair and a desk. Other necessary materials are tongue depressors (Assistant, 4365), stomatoscopes (Hager & Werken, Brillant No. 4, 605400), gloves, and lab coats.

#### Blood collection

Blood samples were taken under medical supervision. Two teams of one nurse and one assistant each were necessary to realize the blood withdrawal of 75 volunteers per day. Blood withdrawal was done following the respective SOP. Therefore, safety needles (Saarstedt, Myltifly Safety, 85.1637.205) and sterile 20 ml syringes (BD, Discardit, 300296) were used. All sample tubes were rinsed according to the respective SOP with 2% nitric acid (Merck, Emsure, 1.00456.2500), methanol (Merck, Emsure, 1.06009.2500), and 18 MΩ ultrapure water (Millipore, Milli-Q Integral 5, ZRXQ005T0) prior to the sampling to avoid any organic and inorganic contamination and were supplemented with heparin (Ratiopharm, Heparin-Natrium-25000, PZN-3029843). Finally 140 ml blood was collected in seven 20 ml syringes and 5 ml were dropped out of the safety needle into a sample tube. The transfer of blood from the syringe into the sampling tubes was done in two laminar flow safety cabinets (Aura Mini, BioAir, LV 30000). Afterwards, samples were immediately transferred into the laboratory for further processing. For safety reasons a set of medical devices, e.g., stethoscope, blood glucose meter, blood pressure meter, and medicine (e.g., physiologic salt solutions, drugs for improved circulation) was assembled in an emergency bag and kept aside ready to use.

#### The laboratory and the sample preparation process

According to international and national biological agents regulation untested human body fluids, such as blood, have to be considered as potentially infectious. In general, the ESB samples are collected from healthy volunteers, not representing any risk group. However, a maximum level of protection would be supplied by a BSL 2 laboratory. Such a laboratory did not exist at each sampling site. Therefore, the necessary infrastructure in the available laboratories was established by IBMT. Following the German regulations with regard to biosafety, general requirements on a biological safety level 2 laboratory are as follows:

Staff members have to wear protective clothing (lab coat and gloves). A personal lock must be provided that has to be equipped with a handsfree sink and disposable towels, an emergency shower, an eye shower, disinfectants and hygiene regulations. The laboratory itself must be a separate room with surfaces easy to clean and resistant against disinfectants. Doors and windows must be closed while working in the laboratory. The entrance door has to show clearly the biohazard sign and should have a window. All processes that may lead to the formation of aerosols must be done in a laminar flow safety cabinet or staff members must wear task specific protective clothing, e.g. goggles, surgical masks, gloves, and lab coats [43].

Blood and 24 h-urine samples were directly processed upon entering the laboratory following the respective SOPs. After the blood samples were transferred into the lab, four of the seven collected blood tubes were directly prepared and packed for freezing. The remaining three blood tubes, each containing 20 ml of blood, were centrifuged for plasma separation (Eppendorf, 5810 R, 5811 000.424). Separation of plasma from the remaining blood cells and portioning into sample tubes was done in a laminar flow safety cabinet (BioAir, EF/S4, H071001) using pipets (Eppendorf, Research, 3120 632.000) with 5 ml pipet tips (Eppendorf, epTIPS, 0030 073.169).

Weight, density, and conductivity of the urine samples were determined in the lab. Therefore, the lab was equipped with an electronic balance (Mettler-Toledo, 11124926), an aerometer (Assisstent, 60008), and a conductivity measurement device (Mettler-Toledo, 51302936). Urine samples were portioned into 9×13 ml (10 ml urine) and 3×30 ml (20 ml urine) cleaned tubes and prepared for freezing. Immediately after sample processing tubes were transferred into a –80°C freezer (Heraeus, HFC586 PLUS-V14, 77710200) and kept there overnight to avoid disruption of the sampling tubes due to a rapid expansion of the freezing liquid. On the following day, samples were transferred into the cryogenic storage vessel (Cryotherm, Bio Safe 420), pre-cooled down to –160°C using liquid nitrogen.

#### Infrastructure, logistics and stuff members

Several instruments were required to conduct the sampling with regard to biosafety regulations and quality assurance. Except of the cryogenic storage vessel for samples and the cryogenic vessel for liquid nitrogen supply which were installed in the transporter, all devices (see table 1) were provided by IBMT and were delivered by a forwarder to each sampling site one day before sampling. At the same day the infrastructure was installed in the rented laboratories of the cooperating institute. All instruments and analytical tools were re-validated and the revalidation was documented in the frame of a quality management system according the GCLP. At least three additional assistants were needed to set up the required infrastructure and to remove it one day after sampling.

**Table 1:** Overview of provided instruments.

Task	Instrument
**Blood withdrawal**	2×Laminar flow cabinet
	2×Treatment bed
**Laboratory (Urine processing)**	1×Aerometer
	1×Electronic balance
	1×Conductivity measurement device
	2×Pipets
**Laboratory (Blood processing)**	1×Laminar flow cabinet
	2×Pipets
	2×Centrifuge
**Freezing and Storing**	1×80°C Freezer
	1×LN tank (150 l)
	1×LN samples storage tank (420 l)

The realization of the sampling event required at least 12 members of staff. Table 2 gives an overview of the required members of staff and their related tasks.

**Table 2:** Overview of required members of staff and their tasks.

Task	Members of stuff
**Reception and data control**	2×Scientists
**Dental fillings**	1×Dentist, 1×Assistant
**Blood withdrawal**	1×Medical Doctor, 3×Assistant
**Laboratory (Urine processing)**	2×Medical Technical Assistant
**Laboratory (Blood processing)**	1×Medical Technical Assistant
**Freezing and Storing**	1×Medical Technical Assistant
	**Total: 12**

#### Generated samples and subsequent analysis

During the sampling procedure different samples were generated depending on their use. Table 3 gives an overview of the generated samples per person. In general, a subset of samples was directly separated for real-time monitoring (RTM) of selected chemicals. After the sampling procedure in 2012 a part of these samples was transferred to the IBMT laboratory to investigate clinical chemical parameters. 24 h-urine samples were thawed and creatinine was measured. Furthermore, creatinine, triclycerides, total protein, and cholesterol were measured in thawed plasma samples. Clinical chemistry of both body fluids was measured using the cobas c111 analyzer (Roche, 04 777 433 001).

**Table 3:** Sample types per Volunteer.

Sample type	Size	Cryo-Repository	Real-Time-Monitoring
**Blood**	20 ml	3	
**Blood**	5 ml		1
**Plasma**	3 ml	7	2
**24** **h-Urine**	10 ml	7	2
**24** **hUrine**	20 ml	3	
	**Total**	**25**	

The other part of the RTM samples was transferred to a respective laboratory that analyzed metal compounds in blood and urine. The results of these studies were published in an annual report to the UBA, brochures and on the homepage of the German ESB (www.umweltprobenbank.de). Each volunteer received a personalized letter with his own results after these RTM analyses were completed. Thereafter, personal data were eliminated and samples were made anonymous according to the German data privacy act for their further storage in the German ESB.

In total, 20 samples per volunteer were cryopreserved and long-time stored immediately after processing and portioning in the laboratory. Considering a maximum of 600 participating volunteers per year, a total of 15.000 subsamples could have been generated. About 3.000 of them got used for real-time monitoring and approximately 12.000 got stored in the German ESB.

## Results and Discussion

### Requirements review of the sampling in 2012

The samplings in 2012 in Muenster (Westphalia), Halle (Saale), Greifswald and Ulm were conducted according to the procedures stated above. The establishment of a quality management system (QMS) following the GCLP has been started by IBMT under these conditions. It has turned out that the varying locations with different standards with respect to laboratory regulations have large implications on the QMS. According to the analysis of process requirements, a mobile laboratory was developed to further optimize, standardize and harmonize these multicenter sampling events and to realize the sampling in 2013 for the first time with a mobile BSL 2 facility.

### Optimization of room layout and workflow

A detailed analysis of the requirements for floor space has been carried out based on the experience of the sampling procedures in 2012. A minimum total floor space of 50 m^2^ is required in a mobile platform to carry out all processes of the workflow (see Table 4).

**Table 4:** Result of requirement analysis for rooms.

Room/compartment	Instruments/furniture	Floor space [m^2^]
**Reception**	Table, chairs (4), kitchenette	10.6
**Privacy compartment** **(2 times)**	Table, chairs (2)	2×1.5
**Waiting area**	Chairs (3)	2.5
**Dental status**	Dentist’s chair, chairs	4.6
**Blood withdrawal and** **initial processing** **(2 times)**	Chair for blood withdrawal,laminar flow cabinet, chairs	2×7.0
**BSL 2 laboratory**		
** - personnel locker room**	Handsfree sink, hand desinfection	2.6
** - main room**	Laminar flow cabinet, centrifuge,cobas C111, pipets,conductivity meter, balance	7.9
** - storage room**	LN tank (150 l), storage tank (420 l)	3.4
**Toilet**		1.1
**Technical compartment**		2.9

A semitrailer with a maximum allowed width and length of 2.5 m by 14 m respectively can thus provide the largest connected floor space (35 m^2^) according to the German road traffic act (special transports with specific allowance and under police control are not considered). Experiences with laboratory vehicles developed in the project “Labor der Zukunft” (www.labor-der-zukunft.com) showed that 32 m^2^ can be realized including all required technical rooms for heating, ventilation and air conditioning. Consequently pull-out extensions have been considered and investigated. A central pull-out compartment of approximately 8 m length was proposed to provide additional space (16 m^2^). Two smaller pull-out compartments (approximately 3 m^2^ each) are located on both sides at the bow of the semitrailer (goose neck).

Urine and blood processing can be carried out in one single laboratory room, but a dedicated workflow has to be implemented to prevent cross interference. In order to separate and localize the sample entrance into the main laboratory, two different material locks were implemented. One lock was specifically designed for six urine containers. The location and use of this lock was designed to enable single-handed delivery of the urine sample into the laboratory.

**Figure 1:**
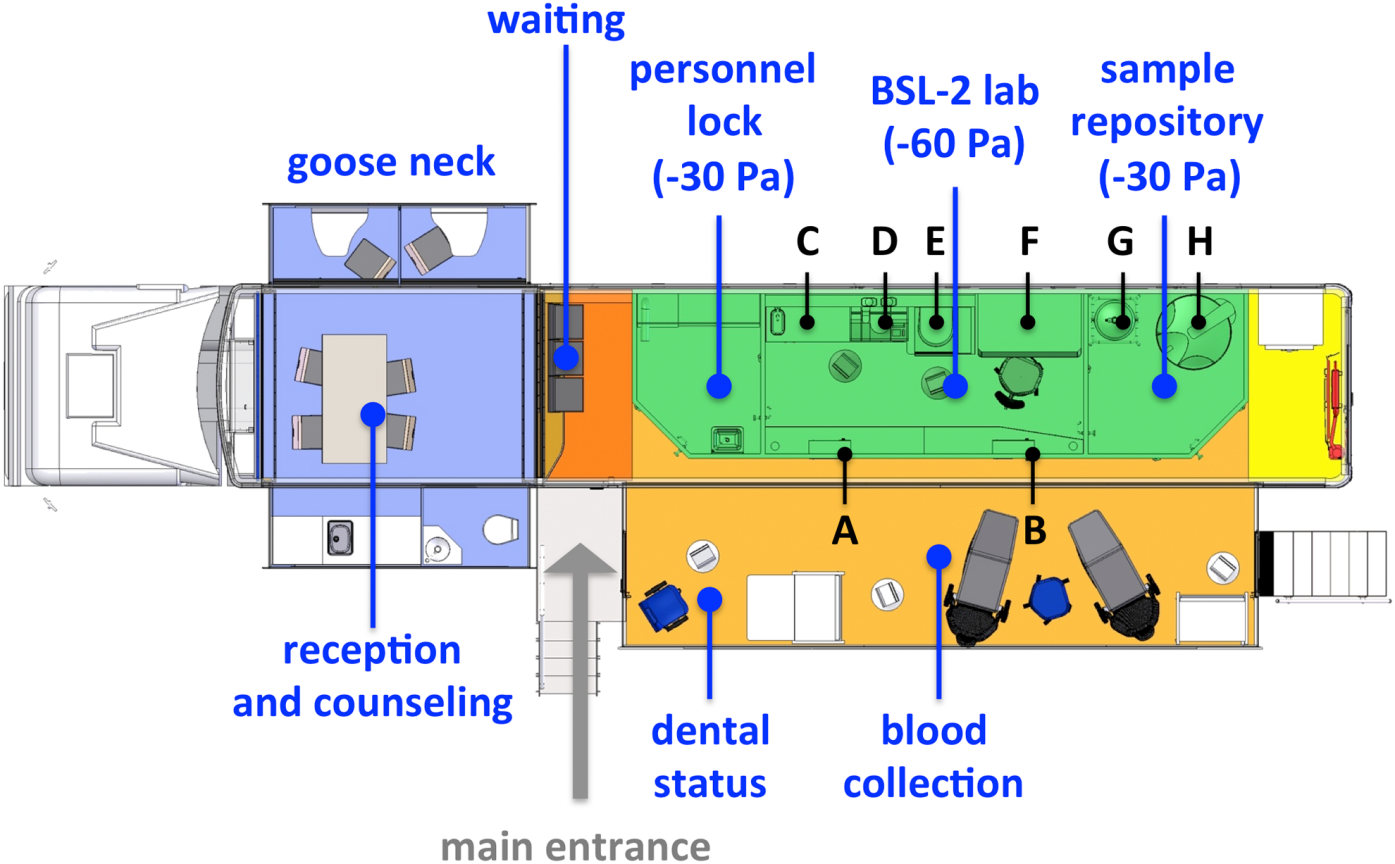
General layout and concept of the mobile epidemiologic laboratory (epiLab). Three motoric driven expansion units provide a reception and counseling area (14.5 m^2^) and an examination room (21.1 m^2^). The laboratory block (15.5 m^2^) has three rooms (personnel lock, BSL-2 lab and sample repository) with individual pressure levels for secure operation.

**Figure 2:**
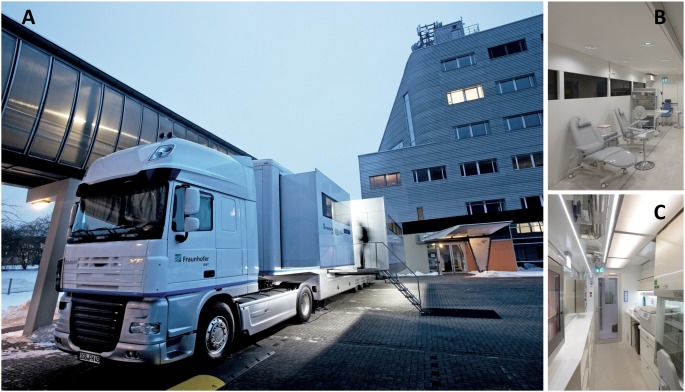
Mobile epiLab at first mission. A: The expandable unit. B: View to the examination room. C: BSL-2 area in the mobile epiLab with urine processing area (background) and blood processing area (foreground).

Figure 1 gives an overview of the developed mobile epidemiologic laboratory (epiLab). As shown, two motoric driven expansible units in the goose neck offer 14.5 m^2^ of room. This part of the trailer is equipped with a sanitary facility, a small kitchen and two small cabins with chairs and folding tables. It is used as reception and counseling unit and allows controlling the self-reported questionnaire of each participant in a private atmosphere. A large motoric driven expansible unit reveals the diagnostic area (21.1 m^2^) and the actual biosafety level 2 laboratory block (15.5 m^2^). The diagnostic area is split into three parts. One part close to the entrance is used as a waiting room, the next part is for the dentist to ascertain dental status (Figure 1, marked *dental status*), and the largest part in the back that is equipped with two laminar flow cabinets and two treatment chairs for blood collection (Figure 1, marked *blood collection*). Figure 1 shows the two material locks in the front of the laboratory block, which allow a direct transfer of urine samples (Figure 1, A) and blood samples (Figure 1, B) into the laboratory. The actual laboratory is organized into three categories: A personnel lock, the main laboratory and the sample repository. A cascade of low pressure with steps of –30 Pa (lock and repository) and –60 Pa respectively (laboratory) is realized with a ventilation system using 100% fresh filtrated air and allowing air renewal rates up to 14 times per hour. Table 5 gives an overview of the different workplaces and instruments that are used during the sampling and which are shown in Figure 1. Figure 2 shows the mobile epiLab at its first mission in Muenster in January 2013.

**Table 5:** Different workplaces and instruments in the laboratory block.

Workspace/Instrument	Letter
**Material lock - Urine**	A
**Material lock - Blood**	B
**Urine/aerometer, balance, conductivity measurement device**	C
**Clinical Chemistry/Roche - Cobas c111 analyzer**	D
**Centrifugation/centrifuge**	E
**Blood processing/laminar flow cabinet**	F
**LN Supply/LN storage tank (Cryotherm - Saturn 300)**	G
**Sample Cryo-preservation/storage tank**	H

### Organization of the general sample procedure and sample processing in the mobile epiLab

**Figure 3:**
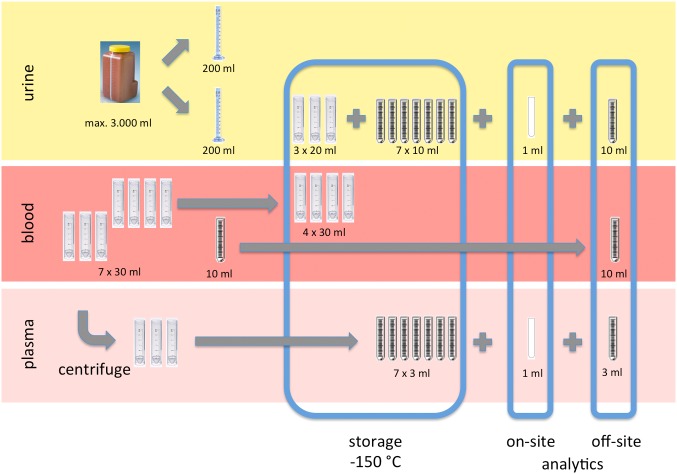
Sample workflow and pre-analytics in the mobile epiLab. Urine and blood samples are processed at the same time and aliquots are stored in the mobile cryo-repository and prepared for off-site analytics. Urine and plasma samples are also analyzed on-site.

In general, after medical history and exposure relevant behavior was recorded and the questionnaire was controlled, the volunteer was asked to place his urine sample into the first material lock (Figure 1, A) by himself. Weight, conductivity and density were measured immediately after the sample entered the lab following the respective SOP. After recording the dental status of the volunteer by a dentist and one assistant, blood was withdrawn in the respective area of the examination room from a medical doctor and one assistant (see Figure 1). Blood samples were immediately placed into the second lock by the assistant (Figure 1, B) and directly processed and prepared for clinical chemistry and cryopreservation. All results were documented following the respective SOP in standardized documentation forms. The procedure applied in 2012 required cooling down samples from body or room temperature to –80°C and later deep freezing to below –140°C in order to prevent the plastic tubes from thermal cracking. With regard to simplifying this process in 2013 a storage vessel with liquid nitrogen cooling was adapted to provide different temperature zones. So, samples can be deep frozen in a gentle two-step process. The Flow chart in Figure 3 describes the sample processing procedure for urine, blood, and plasma samples that is specifically designed for the mobile lab unit and to prevent cross interference.

**Figure 4:**
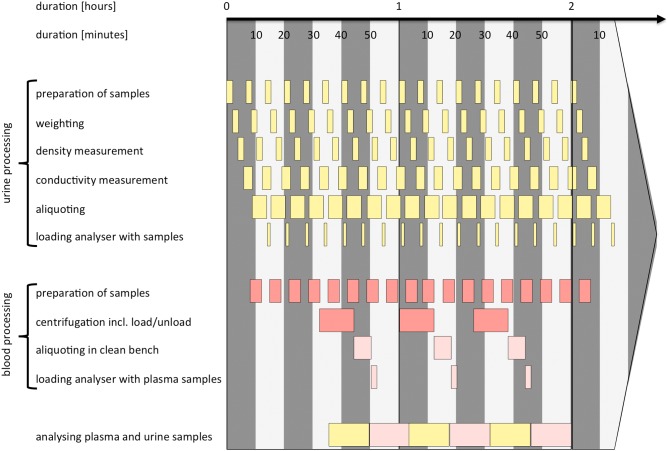
Schematic workflow for the processing of urine (yellow), blood (red) and plasma (pink) samples in the mobile epiLab. Three volunteers per 20 min are assumed for the optimum throughput shown here. The starting delay for the blood processing is caused by the on-site blood draw.

A specific time schedule was set up to manage the required up to 75 volunteers a day and the respective amount of samples and to avoid bottlenecks and waiting periods. A timeframe of 15 to 20 minutes was defined for the realization of all sampling tasks for three volunteers. Figure 4 shows the schedule for each sample type.

### Integration of clinical chemistry

In 2012 clinical chemistry of the body fluids was measured not on site, but in a laboratory at the IBMT headquarter. Therefore, immediately after the sampling, samples were frozen as described in “Materials and Methods” and transferred to the laboratory. Using the mobile laboratory in 2013, for the first time it was possible to integrate the analysis of the described clinical chemical parameters into the sampling process and thus avoiding freezing and transportation of the samples. Hence, blood samples were centrifuged as described. Plasma was separated in the laminar flow safety cabinet and subsequently 1 ml of plasma was pipetted into a respective tube for analysis of clinical chemical parameters using the cobas c111 analyzer (Roche). Urine was homogenized via soft panning and 1 ml of urine was directly pipetted into a respective cobas tube. Thus, results of the analysis for all participating volunteers of each day were available only one hour after the sampling.

### Participants and number of samples in 2012 and 2013

An overview of the acquired volunteers and the actual number of volunteers that participated in 2012 and 2013 for each of the four sampling sites is given in Table 6. As table 6 reveals, using the mobile laboratory and the described workflow, in 2013 the same amount of volunteers and even more were managed within the same time period. Finally, according to the number of volunteers and the number of subsamples described in Table 3, in 2012 a total of 12.650 subsamples from 566 volunteers were generated during the samplings at the four different sampling sites, whereas 12.750 subsamples from 578 volunteers were generated during the samplings in 2013 using the mobile epiLab.

**Table 6:** Overview of acquired and actual participants in 2012 and 2013.

Year	SamplingSite	Date ofsampling	Totalnumber ofacquiredvolunteers	Number ofvolunteersthatparticipated	Number offemalevolunteers	Number ofmalevolunteers	Number offormerparticipations
**2012**	Muenster	20.–21.01.	155	133	63	70	21
	Halle/Saale	26.–27.03.	136	129	64	65	29
	Greifswald	14.–15.04.	135	124	64	60	56
	Ulm	05.–06.06.	140	120	59	61	23
	**Total**		**566**	**506**	**250**	**256**	**129**
**2013**	Muenster	25.–26.01.	153	131	69	62	37
	Halle/Saale	19.–20.03.	141	124	67	57	12
	Greifswald	20.–21.04.	145	131	68	63	53
	Ulm	04.–05.05.	139	124	68	56	17
	**Total**		**578**	**510**	**272**	**238**	**119**

### Sample transport

All samples have to be brought to the central repository in Muenster without breaking the cooling chain**.** Table 7 gives an overview of the distances between the sampling sites and the IBMT headquarter and between the sampling sites and the central repository in Muenster. In 2012, this transport was carried out with a fixed integrated cryotank in a transfer vehicle. Consequently, each sample had to be handled twice for each sampling. In order to avoid these handling processes, in 2013 a removable cryogenic storage vessel has been established in the mobile sampling unit of the epiLab. This tank can be brought, together with all samples, to the central archive in Muenster, unloaded via an included tail lift and connected to the liquid nitrogen supply in the repository.

**Table 7:** Distances of mission locations from headquarter (Sulzbach/Saar) and repository (Muenster).

Mission location	Distance to headquarter [km]	Distance to repository [km]
**Muenster (Westphalia)**	400	0
**Halle (Saale)**	600	400
**Greifswald**	1000	600
**Ulm**	300	600

### The Quality Management System

The German ESB already exists since 1985. Since that time standard operating procedures (SOPs) were established for all relevant steps and were released by the UBA and the German ESB consortium [36]. These SOPs were used to document all the processes in an internal quality management system but not by national or international guidelines.

Experiences made during the sampling events in 2012 were used to define the requirements for a QMS. In a first attempt the existing QMS was converted into a QMS according to GCLP. A QM handbook was written describing all relevant SOPs. It was clearly recognized that the four different sampling sites provide different infrastructures and laboratory settings. Therefore, workflows and processes had to be reorganized for each of the sampling sites preventing a strict standardization of these pre-analytical processes. A QMS according to GCLP was already implemented by Fraunhofer IBMT for their HIV Specimen Cryorepository (HSC) funded by the Bill & Melinda Gates Foundation [44]. These specific guidelines are only internal guidelines for networks funded by the Division of AIDS (DAIDS) of the National Institute for Health (NIH) and do not constitute an international accepted regulation. For this reason, the IBMT is now adapting the QM system to the international DIN EN ISO/IEC 17025 regulation [45], [46] and is aiming an accreditation of the sampling by Germany’s National Accreditation Body (DAkkS). In 2013, with the availability of the mobile laboratory, all processes and workflows related to the sampling event were adapted to the new conditions and standardized. Following the described requirements all SOPs were adapted to the available rooms, workflows and analyses. Finally, the implementation of the mobile lab guarantees the same facility and infrastructure specifically adapted to the need of the sampling procedure of the German ESB. Thus guaranteeing identical treatment of all samples at all sites.

### Stand-alone capabilities

In order to ensure stand-alone operation of the mobile unit and to minimize the requirements for potential locations, the following requirements have been defined:

#### Fresh and waste water

The main requirements resulted from the collection of urine samples. 150 volunteers per sampling can lead to a maximum of 450 l (3 l per volunteer) to be discarded. Assuming a mean value of 1 l of fresh water used per urine sample for flushing, the following minimum requirements for fresh and waste water per mission can be projected: 150 l of fresh water and 600 l of waste water. Including some extra capacities for hand washing and toilet use, a 400 l fresh water tank and a 1.000 l waste water tank are implemented. Both tanks are made of stainless steel and are equipped with electrical heating for frost protection.

#### Heating, ventilation and air condition (HVAC)

In order to cope with the different climate conditions during the mission, the large number of staff and volunteers on board and the regulatory requirements for the laboratory (constant temperature and air exchange), a complex and powerful HVAC system had to be designed and implemented. A water based floor heating driven by an engine block heater (10 kW Diesel) is used to establish basic heating during the winter. The laboratory is supplied with 100% fresh air at an exchange rate of up to 14 times per hour (laboratory only). This fresh air is filtered and conditioned by a central climate system. The target temperature for the incoming air can be set in the range of 20 to 24°C and is specified for ambient temperatures from –10 to 34°C. A low pressure cascade can be established in the laboratory on demand. All secondary rooms are equipped with individual HVAC units with heat pump function for heating.

#### Electrical supply

The entire trailer is designed to be operated with a diesel-based electric generator (34 kVA and 120 l tank). In normal mode, the system lasts for 10 h. An energy management system is implemented to cope with shortages by switching off energy of peripheral systems (lighting, kitchen). Although the generator is acoustically shielded, land line electric power is used for standard operation. A 32 A (CEE) landline is required as a minimum, 6 A (CEE) is recommended to prevent energy management cut-offs and to refresh all internal batteries. The critical laboratory instruments and part of the IT infrastructure are connected to an uninterrupted power supply unit (USV). Via this USV, switching from land line to generator can be done without shortage of electrical energy in the laboratory.

## Conclusions

Since 2013 Fraunhofer IBMT collects up to 15.000 individual human subsamples annually under highly standardized conditions using the introduced mobile laboratory. Clinical chemical parameters like cholesterol, total protein, creatinine, and triglycerides are now analyzed directly on-site. At the same time samples are frozen in the adjacent cryorepository and stored at temperatures below –140°C. Frozen samples are transferred within the vehicle to its final destination without breaking the cooling chain. Since all processes are conducted following ESB specific SOPs according to the DIN EN ISO/IEC 17025 regulation, the quality of each sample is guaranteed. Finally, difficulties in the process of standardization resulting from different sampling sites are avoided and the collected samples are kept ready, characterized and free of change for retrospective analyses in their final archive, the German ESB.

Lab settings and analysis tools in the Fraunhofer IBMT mobile laboratory can be changed and modified. With an adapted QM system this mobile lab allows the standardization of every multicenter sampling event in the field of human related research, scientific surveys, and clinical trials.
